# Supplementary material for: The causal relationship between the human gut microbiota and pyogenic arthritis: a Mendelian randomization study
Source: Front Cell Infect Microbiol. 2024 Nov 26;14:1452480. doi: 10.3389/fcimb.2024.1452480 (PMC11629706; doi:10.3389/fcimb.2024.1452480)
Supplement: Supplementary file 1 [file DataSheet1.zip › mendelian test/ebi-a-GCST90027477.csv_forest.pdf]

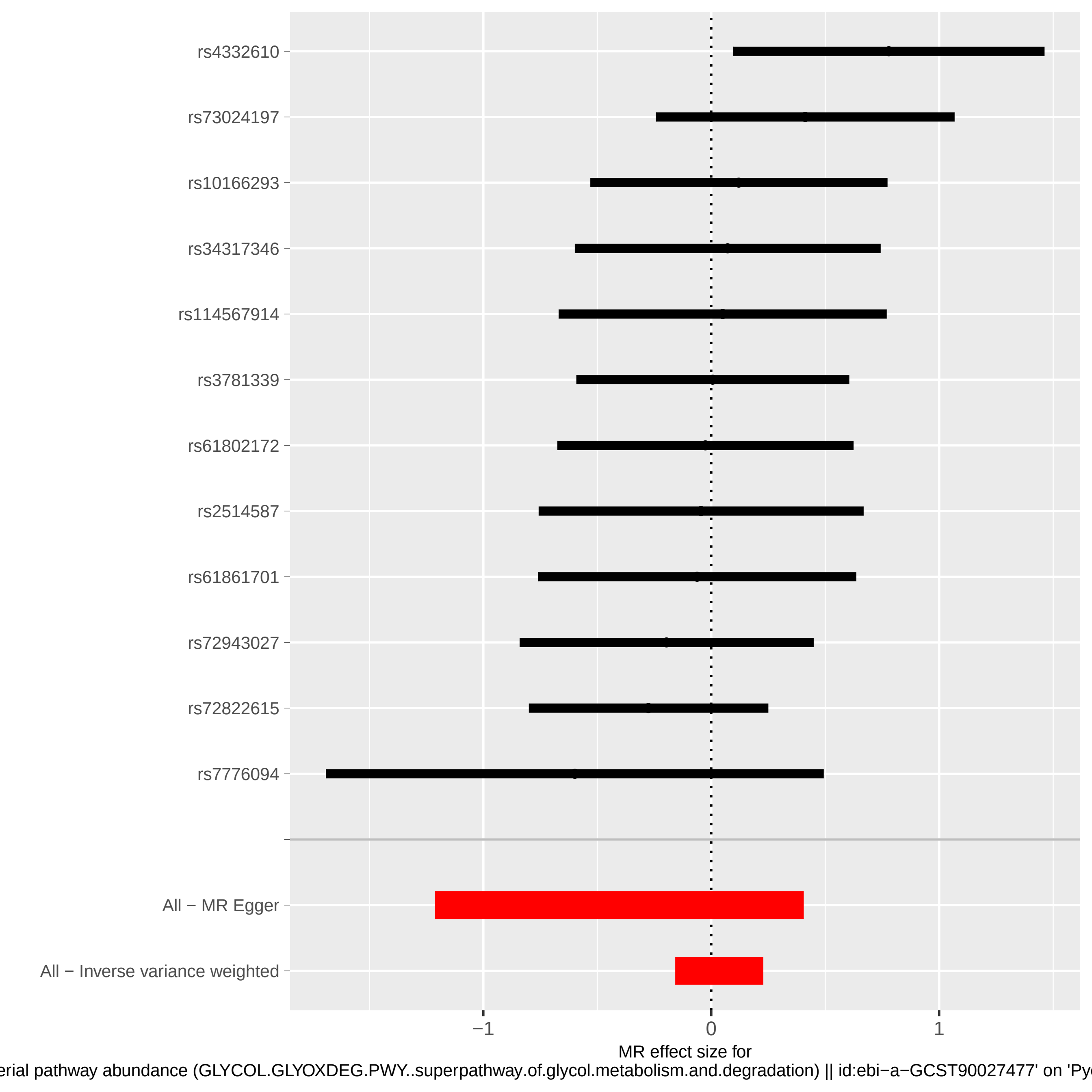
rs4332610
rs73024197
rs10166293
rs34317346
rs114567914
rs3781339
rs61802172
rs2514587
rs61861701
rs72943027
rs72822615
rs7776094
All − MR Egger
All − Inverse variance weighted
−1
0
1
MR effect size for
erial pathway abundance (GLYCOL.GLYOXDEG.PWY..superpathway.of.glycol.metabolism.and.degradation) || id:ebi−a−GCST90027477' on 'Py
